# Political participation in an electoral autocracy: latent profiles of efficacy, interest, ideology, and trust in Hungary

**DOI:** 10.3389/fpsyg.2026.1856578

**Published:** 2026-07-30

**Authors:** Málna Anna Benza, Zsolt Péter Szabó, Sointu Leikas, Jan-Erik Lönnqvist

**Affiliations:** 1 Doctoral School of Psychology, ELTE Eötvös Loránd University, Budapest, Hungary; 2 Social Psychology Department, Institute of Psychology, ELTE Eötvös Loránd University, Budapest, Hungary; 3 Department of Psychology, Institute of Strategy and Management, Corvinus University of Budapest, Budapest, Hungary; 4 Helsinki Institute of Social Sciences and Humanities, University of Helsinki, Helsinki, Finland; 5 Swedish School of Social Science, University of Helsinki, Helsinki, Finland

**Keywords:** democratic backsliding, electoral autocracy, institutional trust, political efficacy, political ideology, political interest, political participation

## Abstract

Hungary is often portrayed as an exceptional case of democratic backsliding driven by unique political and cultural conditions. We tested this view by analyzing five waves of European Social Survey data from Hungary (2014–2023) and conducting latent profile analyses based on political interest, political self-efficacy, institutional trust, and ideological self-placement. Across time, four recurring profiles emerged—Moderate system believers, Committed Fidesz voters, Alienated citizens, and Diverse opposition members—with a fifth profile, the Uncommitted opposition, appearing in 2020 and 2023. These profiles do not support a simple picture of Hungarian society as rigidly polarized or cleanly divided into tribal political camps. Instead, they reveal a fragmented and fluid political landscape in which support for the government, opposition, and disengagement take multiple psychological forms, with distinct patterns of institutionalized and non-institutionalized political participation. The findings suggest that democratic backsliding in Hungary is not only a product of exceptional leadership or national idiosyncrasy, but may reflect broader dynamics that can emerge wherever alienation, fatigue, and low efficacy erode democratic engagement. More broadly, the study raises questions about how scholarly narratives of democratic decline are constructed, including how researcher positionality may shape the interpretation of heterogeneous empirical patterns.

## 1 Introduction

Hungary is one of the most widely discussed European cases of democratic backsliding. During the period of Fidesz rule under Viktor Orbán, the country underwent substantial institutional transformation that limited the ability of opposition parties and dissatisfied citizens to influence politics (Enyedi and Krekó, 2018). These macro-level changes have been extensively studied, although there remains debate about why democratic backsliding occurred, how exactly it unfolded, and why it became politically sustainable. Fidesz capitalized on the collapse of the previous social democratic government and the aftermath of the 2008-2009 global financial crisis to secure a supermajority in 2010, which enabled far-reaching restructuring of the political system (Csehi, 2019; Enyedi and Krekó, 2018; Sonnevend and Kövesdi, 2023; Szebeni and Salojärvi, 2022). During the period examined in this study, Fidesz combined conservative nationalism, with an emphasis on traditional family values and national sovereignty, alongside opposition to migration and liberal rights agendas (Lönnqvist et al., 2021). Scholars have classified the resulting regime in different ways, including as an illiberal democracy (Lönnqvist et al., 2021), an electoral or competitive autocracy (Levitsky and Way, 2020; Liboreiro and Zsiros, 2022), and an informational autocracy (Guriev and Tresiman, 2019). In the present study, we use the term electoral autocracy to refer to a regime type in which elections continue to take place, but meaningful political competition and citizen influence are constrained by weakened checks and balances, uneven access to institutional resources, restricted media pluralism, and the erosion of democratic accountability (Levitsky and Way, 2020; Liboreiro and Zsiros, 2022). In such a context, citizens may formally retain opportunities for electoral participation, while at the same time perceiving that their capacity to influence political outcomes is limited. This may be especially relevant for citizens who do not sympathize with the incumbent regime, for whom electoral and non-electoral forms of participation may appear less capable of producing meaningful political change.

Compared with the extensive literature on Hungary’s macro-level institutional transformation, less is known about how citizens’ individual-level psychological-political orientations were configured during this period, particularly with respect to political interest, political efficacy, institutional trust, and ideology. This does not mean that such questions have been entirely neglected. Rather, the available evidence remains comparatively scattered, especially when it comes to how these orientations combine into recurring citizen profiles across time. One plausible expectation is that prolonged elite polarization would produce a deeply divided society in which citizens align into rigid camps of government supporters, opponents, and disengaged bystanders (Bíró-Nagy et al., 2022). In line with this view, Hungary has been described as a case of political tribalism, in which elite conflict fosters social divisions between perceived “good” and “evil” camps (Krekó, 2021). Yet the existing evidence complicates this picture. Scoggins (2022) found that economic satisfaction predicted voting for Fidesz more strongly than ideological alignment, suggesting that support for the government was not reducible to tribal loyalty. Similarly, Wunsch and Gessler (2023) argued that Fidesz’s success rested on a heterogeneous coalition rather than a monolithic populist voter base. Research on Hungarian political values and identity patterns has also pointed to a more complex structure of political attitudes than a simple binary division would imply: Szabó et al. (2021), for example, found strong party-based differences in institutional trust and satisfaction with democracy, but also broad support for democracy across political camps and a substantial centrist or moderate component in ideological self-placement. Moreover, recent work on political participation emphasizes that citizens may differ not only in whether they participate, but also in how they interpret different acts as political participation in the first place (Déri and Szabó, 2025). Even Krekó’s (2021) findings suggested that most respondents rejected extreme tribalist positions and recognized the legitimacy of dissenting voices. Together, these studies raise the possibility that political alignment in Hungary was more heterogeneous than binary accounts of polarization imply.

This heterogeneity is particularly relevant for understanding political participation, which is the main outcome of interest in the present study. In an electoral autocracy, where citizens formally retain opportunities for participation but may doubt their ability to influence outcomes, participation may depend not only on party alignment, but also on broader configurations of political interest, efficacy, trust, and ideology. In the present study, we therefore focus on two broad forms of legal political participation measured in the European Social Survey (ESS): institutionalized participation, especially voting, and legal non-institutionalized participation, such as peaceful protest and other lawful forms of collective action (Marien et al., 2010). We examine how these forms of participation are related to latent profiles based on four theoretically relevant psychological-political orientations: political interest, political self-efficacy, institutional trust, and left–right ideological self-placement.

In democratic contexts, political interest and political self-efficacy are well-established predictors of participation: citizens who are more interested in politics and feel more capable of influencing political outcomes are generally more likely to engage (Amnå and Ekman, 2014; Blais, 2006; Dostie-Goulet, 2009; Reichert, 2018; Verba et al., 1995). Institutional trust has a more complex relationship with participation. Some degree of trust may be necessary for conventional participation to retain meaning (Almond and Verba, 1963), but distrust may also motivate protest and other non-institutionalized forms of action (Kaase, 1999; Hooghe and Marien, 2013). Marien and Christensen (2013) similarly argued that the relationship between trust and participation depends on the form of participation: trust is more closely associated with institutionalized participation, whereas distrust may encourage non-institutionalized, elite-challenging forms of action. Ideology may also shape participation; in the present study, ideology is understood in the limited sense of citizens’ left–right self-placement as measured in the ESS. References to polarization therefore concern ideological distance or extremity on this same left–right dimension, rather than a broader account of ideological content. Such polarization may mobilize citizens by increasing the perceived stakes of politics, but it may also demobilize moderates and politically unaffiliated individuals (Hetherington, 2001; Levendusky, 2010; Rogowski, 2014), although recent meta-analytic evidence suggests that individual-level ideological polarization is positively associated with both electoral and non-electoral participation (Kołczyńska, 2025).

These relationships may take a distinctive form in a system marked by democratic erosion. In non-democratic settings, efficacy may matter less as a belief in direct influence and more as a belief that one can contribute to a broader movement (Ayanian et al., 2021). In Hungary, repeated institutional changes favoring the dominant party may also have weakened efficacy among supporters of smaller parties, while polarization may have further reduced efficacy among citizens without strong party ties (Karp and Banducci, 2008). Likewise, in post-communist contexts, low institutional performance can erode trust, weaken civil society, and discourage political engagement (Mieriņa, 2014; Ejrnæs, 2017). However, distrust does not necessarily lead to disengagement: when combined with political interest and a sense of efficacy, it may also support protest or other non-institutionalized forms of participation. These considerations suggest that political interest, efficacy, trust, and left–right ideological self-placement remain relevant for understanding participation in Hungary, but that they may combine in ways that are not fully captured by variable-centered approaches.

To address this issue, we adopt a person-centered approach and use latent profile analysis (LPA). Unlike variable-centered methods, which estimate associations across the full sample, person-centered approaches are designed to identify subgroups of individuals characterized by distinct configurations of attitudes and beliefs (Meyer et al., 2013; Stanley et al., 2016). Relatedly, Christensen (2016) showed, using cluster analysis rather than LPA, that different combinations of political support and subjective political empowerment had different implications for protest participation, illustrating the relevance of configurational approaches to political dissatisfaction and participation. LPA is particularly useful for the present study because our aim is not only to examine whether political interest, efficacy, institutional trust, and ideology are associated with participation on average, but also to investigate whether these orientations combine into distinct citizen profiles with different patterns of electoral and non-institutionalized participation. Recent work has demonstrated the value of person-centered approaches in political psychology for capturing meaningful heterogeneity in political attitudes and behavior (Lönnqvist et al., 2025).

Although the distinction between profile indicators and correlates is partly open to debate, our choice reflects the substantive aim of the study. We treat political interest, political self-efficacy, institutional trust, and left–right ideological self-placement as profile indicators because they capture citizens’ foundational orientations toward the political system and their perceived place within it. Political participation is then treated as the main outcome of interest: we examine whether these profiles differ in voting and legal non-institutionalized participation.

Additional variables are included to clarify the meaning of the profiles. Satisfaction with government and democracy is included to assess whether the profiles also differ in broader evaluations of the political system, a dimension closely related to political support and system justification (Szabó and Lönnqvist, 2021). Happiness is included as a general indicator of subjective well-being, because system justification theory suggests that support for the status quo may have a palliative function for well-being (Jost and Hunyady, 2003). These variables are therefore not treated as primary outcomes, but as secondary correlates that help clarify whether the profiles differ not only in participation, but also in broader regime evaluations and subjective well-being. Attitudes toward European integration are included because Fidesz’s politics has been strongly organized around a sovereignty-based critique of the European Union, rather than simple support for or rejection of EU membership (Hargitai, 2020). Finally, demographic characteristics are used to examine the social bases of the profiles, an opportunity made possibly by the representative nature of the ESS data.

Accordingly, the present study asks two main questions. First, what recurring citizen profiles can be identified in Hungary based on political interest, political self-efficacy, institutional trust, and left–right ideological self-placement? Second, how do these profiles differ in institutionalized and non-institutionalized political participation? Using latent profile analysis on data from rounds 7 (2014/15), 8 (2016/17), 9 (2018/19), 10 (2020/21), and 11 (2023/24) of the ESS, we examine whether Hungarian citizens during this period of Fidesz rule were best characterized by the expected categories of government supporters, opponents, and disengaged bystanders, or whether their psychological-political orientations formed more heterogeneous configurations. Based on previous work on elite polarization and political tribalism, we expected to identify at least one politically engaged pro-government profile, one or more oppositional profiles characterized by lower trust in the system, and a more disengaged profile marked by low interest, low efficacy, and low trust. We then examined whether these profiles differed in voting and legal non-institutionalized participation, while also considering their associations with satisfaction, happiness, EU attitudes, and demographic characteristics.

## 2 Method

### 2.1 Participants and procedure

We used data from five rounds of the ESS, conducted biennially between 2014 and 2023. The ESS data are openly accessible and were downloaded from the ESS data portal^1^ in May 2025. Our sample consisted of Hungarian respondents who provided a response to at least one of the 10 items used as indicators in the latent profile analysis. The sample sizes for each round were as follows: *N*_2014_ = 1,697, *N*_2016_ = 1,614, *N*_2018_ = 1,661, *N*_2020_ = 1849, and *N*_2023_ = 2,118. These rounds were selected based on two main criteria: first, Round 7 (2014) marked the introduction of the current set of variables, which were subsequently used consistently in late rounds; second, 2014 is also notable as the year when Viktor Orbán publicly declared Hungary to be an illiberal democracy.

### 2.2 Measures

#### 2.2.1 Latent profile analysis indicators

For the LPA, 10 items measuring participants’ system-related attitudes and beliefs were selected. These items were consistently measured across all four rounds of the ESS and reflected political interest, internal political efficacy, institutional trust, and left–right ideological self-placement. The items included: (1) *How interested would you say you are in politics?* (Polintr; *1 = Very interested; 4 = Not at all interested*), this item was reverse-coded for easier interpretation; (2) *How much would you say the political system in Hungary allows people like you to have a say in what the government does?* (AlSay; *1 = Not at all; 5 = Very much*); (3) *How able do you think you are to take an active role in a group involved with political issues?* (ActRole; *1 = Not at all able; 5 = Completely able*); (4) *How much would you say that the political system in Hungary allows people like you to have an influence on politics?* (AlInfl; 1 = Not at all; 5 = A great deal); (5) *How confident are you in your own ability to participate in politics?* (Conf; *1 = Not at all confident; 5 = Completely confident*); (6) *How much do you personally trust each of the following institutions: the legal system?* (TrLgl); (7) *politicians?* (TrPol); (8) *political parties?* (TrPart); (9) *Hungary’s parliament?* (TrParl; items 6 to 9 were all responded with the scale 0 = No trust at all to 10 = Complete trust); and (10) *In politics people sometimes talk of “left” and “right.” Where would you place yourself on this scale, where 0 means the left and 10 means the right?* (L-R; *0 = Left; 10 = Right*). Descriptive statistics for the 10 indicators are presented in Table 1.

**Table 1:** Sample means (and standard deviations) of the LPA indicators and conditional covariates, and descriptives of categorical covariates.

	Round 7 (2014) *N* = 1,697	Round 8 (2016) *N* = 1,614	Round 9 (2018) *N* = 1,661	Round 10 (2020) *N* = 1849	Round 11 (2023) *N* = 2,118
LPA indicators					
Political interest					
1. Polintr	1.96 (0.86)	2.06 (0.89)	1.98 (0.83)	1.99 (0.81)	2.12 (0.89)
Political efficacy					
2. AlSay	2.37 (2.30)	2.02 (0.90)	2.00 (0.94)	2.02 (0.93)	1.96 (0.95)
3. ActRole	2.22 (2.39)	1.97 (1.02)	1.87 (0.98)	1.83 (0.94)	1.88 (0.97)
4. Allnfl	2.18 (2.19)	1.96 (0.86)	1.94 (0.90)	1.91 (0.87)	1.88 (0.89)
5. Conf	2.56 (2.48)	1.91 (0.97)	1.78 (0.94)	1.76 (0.92)	1.86 (0.97)
Institutional trust					
6. TrLgl	4.62 (2.55)	5.43 (2.58)	5.52 (2.65)	5.44 (2.40)	5.36 (2.70)
7. TrPol	2.93 (2.48)	3.73 (2.57)	3.93 (2.56)	4.17 (2.55)	4.10 (2.67)
8. TrPart	2.95 (2.41)	3.57 (2.48)	3.78 (2.47)	4.11 (2.47)	4.04 (2.63)
9. TrParl	3.85 (2.52)	4.53 (2.58)	4.54 (2.66)	4.68 (2.57)	4.56 (2.87)
Political ideology					
10. Left–Right	5.32 (2.32)	5.84 (2.52)	5.35 (2.53)	5.79 (2.41)	5.66 (2.72)
Covariates					
Age (years)	49.9 (18.3)	50.8 (18.8)	51.0 (18.5)	50.5 (18.8)	50.6 (18.5)
Gender (% women)	57.5%	58.1%	57.5%	62.2%	60.6%
Voted in last election (% voted out of those eligible)	69.1%	68.7%	69.0%	70.0%	77.9%
At least 1 collective action during last 12 months	14.7%	11.9%	10.6%	10.0%	14.5%
Happiness	6.37 (2.17)	6.81 (2.08)	6.64 (2.08)	7.02 (1.97)	7.10 (1.99)
Satisfaction with government	3.52 (2.48)	4.59 (2.57)	4.48 (2.73)	4.83 (2.79)	4.42 (2.78)
Satisfaction with democracy	4.10 (2.48)	4.83 (2.57)	4.47 (2.70)	5.05 (2.75)	4.45 (2.74)
EU unification	4.68 (2.47)	4.12 (2.69)	4.57 (2.60)	5.23 (2.43)	4.61 (2.72)

Political interest (polintr) was measured on a scale from 1 to 4 (the item was reversed so that higher values mean more political interest), political efficacy items were measured on a scale from 1 to 5, and institutional trust items as well as the Left–Right placement were measured on a scale from 0 to 10. Higher values on the Left – Right scale refer to being more on the political right. Happiness, Satisfaction with government, Satisfaction with democracy, and EU unification attitude were rated on a scale from 0 to 10 and this original scale was used.

#### 2.2.2 Covariates

In addition to the 10 LPA indicators listed, several covariates were extracted from the ESS datasets. The main covariates of interest were the political participation measures, including voting behavior and participation in non-institutionalized collective action. Additional secondary covariates were included to help interpret the obtained profiles, including demographic data, levels of happiness, satisfaction with government and democracy, and attitudes toward European integration. Descriptive statistics for the covariates are presented in Table 1.

##### 2.2.2.1 Voting behavior

Voting behavior was assessed using two questions. First, participants were asked: *Some people do not vote nowadays for one reason or another. Did you vote in the last Hungary’s national election?* with three response options: *1 = Yes, 2 = No*, and *3 = Not eligible to vote*. The third category was excluded from the analyses. Participants who voted were then asked: *Which party did you vote for in that election?* with each candidate party in the given election listed as choice options (see Supplementary Table S1 for a breakdown of voting behavior).

##### 2.2.2.2 Non-institutionalized collective action

To obtain an indicator of participants’ participation in non-institutionalized collective action, responses to the question *There are different ways of trying to improve things in Hungary or help prevent things from going wrong. During the last 12 months, have you done any of the following?* were utilized. This prompt was followed by a series of non-institutionalized collective action (CA) items. The number and content of these items varied slightly between the four rounds. The 5 CA items that were identical across the four rounds were used (*contacted a politician, government or local government official*; *worn or displayed a campaign badge/sticker*; *signed a petition; took part in a public demonstration* and *boycotted certain products*). These items were answered on a Yes/No scale. As the overall level of collective action was low at each round (see Table 1), a binary variable indicating whether the respondent had conducted any collective action (out of the 5 actions listed above) vs. none during the last 12 months was created.

##### 2.2.2.3 Demographics

Self-reported gender, age, years of education, and domicile (type of residence) were included. Gender was coded as a binary male/female variable, while age and education were used as continuous variables. Domicil was a 5-level categorical variable with the levels *big city*, *suburb or outskirts of a big city*, *small town*, *country village*, and *farm or countryside home*. Descriptive statistics for demographic variables are shown in Table 1, except for domicile, which is reported in Supplementary Table S3.

##### 2.2.2.4 Happiness and satisfaction with institutions

The items *How happy are you* (*0 = Extremely unhappy*; *10 = Extremely happy); How satisfied you are with the national government (0 = Extremely dissatisfied; 10 = Extremely satisfied); How satisfied you are with the way democracy works in Hungary (0 = Extremely dissatisfied; 10 = Extremely satisfied)* were obtained from the ESS datasets.

##### 2.2.2.5 Attitudes toward European Union unification

Attitudes toward European Union unification was measured with one item: *Should European unification go further or has it gone too far (0 = Unification has already gone too far; 10 = Unification should go further).*

## 3 Results

Latent profile analyses (LPA) were conducted with MPlus statistical software version 8.9 (Muthén and Muthén, 2017). Indicators were standardized prior to the analyses to facilitate convergence and interpretation. A classic LPA approach was applied, where all indicators were set as uncorrelated within classes. Full information maximum likelihood estimation was used to handle missing data, and parallel starts were utilized to ensure obtaining global maximum likelihood solution. Two to seven class solutions were investigated, and Akaike’s Information Criterion (AIC), Bayesian Information Criterion (BIC), sample-size adjusted BIC, bootstrapped likelihood ratio test (BLRT), Vuong-Lo–Mendell-Rubio likelihood ratio test, bootstrapped likelihood ratio test (BLRT), entropy, and interpretability were used as criteria for choosing the number of latent classes. The LPAs were conducted separately for different ESS rounds.

Table 2 presents the fit indices and likelihood ratio test results for the 2- to 7-class solutions. For the 2014 data, the 6-class solution produced one class with *N* = 3, so the 7-class solution was not tested for that round. First, all solutions for all rounds had acceptable entropy (>0.80). Second, BLRT could not distinguish between solutions. Third, the fit indices diminished for each added class, but the drops became smaller after four classes. The Vuong-Lo–Mendell-Rubio likelihood ratio test became non-significant after four classes for 2014 and 2016 data, and after five classes for 2018, 2020, and 2023 data. Overall, statistical indicators suggested 4- or 5-class solutions, without a strong preference between the two across rounds. However, to ensure theoretical interpretability, we also considered conceptual clarity, ensuring that the final solution did not include multiple classes with little differentiation (Yip et al., 2025). Based on statistical findings and theoretical considerations, 4-class solutions were selected for 2014, 2016 and 2018 data, while a 5-class solution was chosen for the 2020 and 2023 data. Diagonal classification probabilities for the chosen solutions ranged from 0.87 to 0.96.

**Table 2:** Results of latent profile analyses: fit indices and likelihood ratio test results for 2–7 class solutions.

Round	AIC	BIC	SABIC	V-L-M-R	BLRT	Entropy
2014 (round 7)						
2 classes	41627.7	41796.3	41697.8	*p* < 0.0001	*p* < 0.0001	0.90
3 classes	40585.0	40813.3	40679.9	*p* = 0.004	*p* < 0.0001	0.88
4 classes	39412.7	39700.8	39532.5	*p* = 0.002	*p* < 0.0001	0.88
5 classes	38866.4	39214.4	39011.0	*p* = 0.072	*p* < 0.0001	0.86
6 classes	38757.2	39165.0	38926.7	*p* = 0.438	*p* < 0.0001	0.88
7 classes	38076.5	38544.0	38270.8	*p* = 0.491	*p* < 0.0001	0.87
2016 (round 8)						
2 classes	39415.4	39582.4	39483.9	*p* = 0.001	*p* < 0.0001	0.86
3 classes	38113.6	38339.9	38206.4	*p* = 0.006	*p* < 0.0001	0.84
4 classes	37057.9	37343.4	37175.0	*p* = 0.001	*p* < 0.0001	0.86
5 classes	36622.1	36966.9	36763.6	*p* = 0.070	*p* < 0.0001*	0.84
6 classes	36275.3	36679.3	36441.0	*p* = 0.059	*p* < 0.0001	0.85
7 classes	36017.8	36481.0	36207.8	*p* = 0.619	*p* < 0.0001	0.85
2018 (round 9)						
2 classes	41503.1	41671.0	41572.5	*p* < 0.0001	*p* < 0.0001	0.85
3 classes	40036.6	40264.1	40130.7	*p* < 0.0001	*p* < 0.0001	0.84
4 classes	39087.4	39374.4	39206.0	*p* = 0.0001	*p* < 0.0001	0.85
5 classes	38403.7	38750.3	38547.0	*p* = 0.0004	*p* < 0.0001	0.84
6 classes	38081.6	38487.8	38249.5	*p* = 0.308	*p* < 0.0001	0.83
7 classes	37686.9	38152.6	37879.4	*p* = 0.337	*p* < 0.0001	0.84
2020 (round 10)						
2 classes	45949.9	46121.1	46022.6	*p* < 0.0001	*p* < 0.0001	0.86
3 classes	44270.5	44502.4	44369.0	*p* = 0.007	*p* < 0.0001	0.85
4 classes	43482.8	43775.5	43607.1	*p* = 0.075	*p* < 0.0001	0.85
5 classes	42357.9	42711.4	42508.0	*p* < 0.001	*p* < 0.0001	0.87
6 classes	41727.5	42141.7	41903.4	*p* = 0.203	*p* < 0.0001	0.88
7 classes	41427.0	41901.9	41628.7	*p* = 0.705	*p* < 0.0001	0.85
2023 (round 11)						
2 classes	52432.5	52607.9	52509.4	*p* < 0.0001	*p* < 0.0001	0.88
3 classes	50037.5	50275.1	50141.7	*p* < 0.001	*p* < 0.0001	0.88
4 classes	48734.2	49034.1	48865.7	*p* < 0.001	*p* < 0.0001	0.88
5 classes	48054.0	48416.1	48212.8	*p* = 0.012	*p* < 0.0001	0.86
6 classes	47437.8	47862.2	47623.9	*p* = 0.579	*p* < 0.0001	0.86
7 classes	47076.9	47563.5	47290.3	*p* = 0.195	*p* < 0.0001	0.85

AIC, Akaike’s Information Criterion; BIC, Bayesian Information Criterion. SABIC, sample-size adjusted BIC; V-L-M-R, Vuong-Lo-Mendel-Rubio likelihood ratio test for *k* vs. *k* − 1 classes; BLRT, bootstrapped likelihood ratio test for *k* vs. *k* − 1 classes. *Best loglikelihood value in BLRT failed to replicate even with random starts of 500 and 200; thus, the *p*-value cannot be considered reliable.

**Figure 1:**
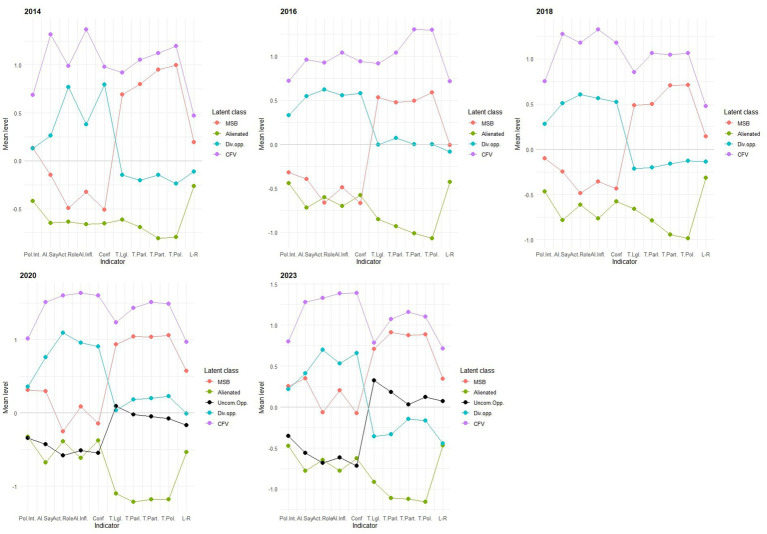
Levels of indicators by class for each round.

Figure 1 illustrates the levels of indicators for the chosen class solution in each round. The indicators were standardized, with zero representing the sample mean.

Next, proportions of voters of different parties among different latent class members were investigated to enhance the interpretability of the class structure. Supplementary Table S1 shows sample voting behavior per round, and Supplementary Table S2 presents the voting behavior of different classes. Based on the LPA and voting behavior, we concluded that roughly three similar classes emerged consistently across all rounds.

We labeled one class as Moderate system believers, a relatively large class characterized by centrist political views, moderate political interest, high trust in institutions, and low political efficacy. This class consistently voted for Fidesz more often than other classes. The size of this class, based on most likely class membership, was *N* = 301 (18% of the sample), 341 (21%), 429 (26%), 314 (17%), and 358 (17%) for 2014, 2016, 2018, 2020, and 2023, respectively (note that the class sizes from 2020 and 2023 cannot be directly compared to the class sizes from 2014–2018 due to different number of classes).

A second class, labeled the Committed Fidesz voters was a smaller group characterized by right-wing political views, exceptionally high political interest, strong institutional trust, and high political efficacy. The majority of this class voted for Fidesz, with very few non-voters. The size of this class was *N* = 298 (18%), 285 (18%), 283 (17%), 118 (6%), and 332 (16%), respectively.

A third class, the Alienated was marked by moderately left-wing views, low political interest, low institutional trust, and very low political efficacy. Members of this class were more likely to abstain from voting, and when they did vote, they were less inclined to support Fidesz. This class was fairly large, with the size of *N* = 709 (42%), 552 (34%), 580 (35%), 459 (25%), and 590 (28%), respectively.

A four, less clearly defined class was the Diverse opposition. This group held centrist-left political views, had average to high political interest, low institutional trust, and relatively high political efficacy. In 2014, they rarely voted for Fidesz, but in subsequent rounds, their voting behavior became more mixed, with some members supporting Fidesz at an average frequency. This internal heterogeneity was reflected in their opposition to the political establishment, as they showed relatively high rates of voting for both the MSZP (the social-democratic party) and Jobbik (the radical right-wing party), while also including members who supported Fidesz in later rounds. The size of this class was *N* = 389 (23%), 436 (27%), 369 (22%), 371 (20%), and 385 (18%), respectively.

The additional class in 2020 and 2023 was labeled Uncommitted opposition. This group was characterized by a slightly left-leaning political ideology, low political interest, moderate institutional trust, and low political efficacy. Members of this class were more likely to vote than those in the ‘Alienated’ class, and their voting behavior roughly mirrored that of the broader sample. This class had *N* = 587 (32%) in 2020 and 453 (21%) in 2023.

Next, the BCH method (e.g., Vermunt, 2010) was used to investigate the relationships between class membership and continuous and binary covariates. This analytical technique investigates the relationships between established classes and external variables by assigning weights reflecting the measurement error inherent in most likely latent class membership. This approach preserves the unconditionally established class structure while controlling for measurement error in class membership. Differences between classes are investigated via chi-square tests in this approach.

**Figure 2:**
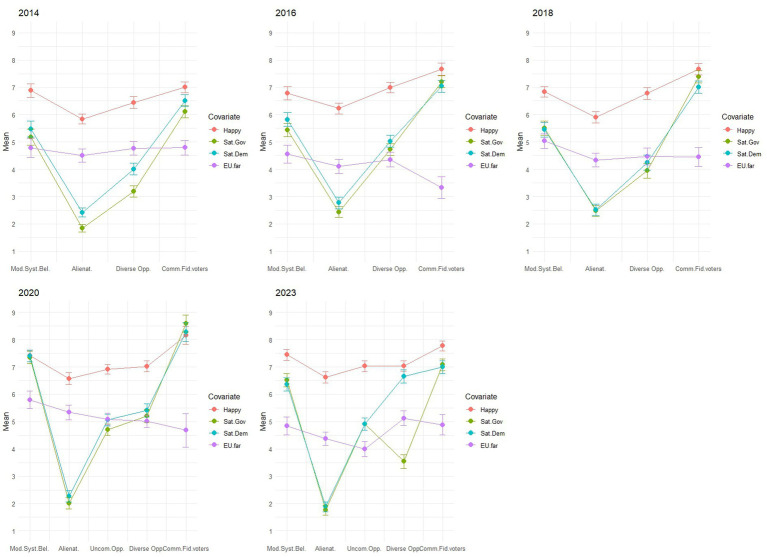
Covariate means per class and round.

The results for continuous covariates are shown in Table 3 and Figure 2. As shown there, Committed Fidesz voters were generally happier and more satisfied with the government and democracy compared to other classes, though Moderate system believers exhibited similar levels of satisfaction. Members of the Alienated class were consistently less happy and less satisfied than all other groups. Regarding attitudes toward EU unification, no strong between-class differences emerged overall. Moderate system believers were somewhat more positive than other classes in 2018 and 2020, whereas in 2023 the most positive attitudes were observed among Diverse Opposition members.

**Table 3:** Results of Chi-square tests of between-class differences for continuous covariates.

	2014	2016	2018	2020	2023
	χ^2^	χ^2^	χ^2^	χ^2^	χ^2^
Happiness					
MSB vs. alienated	39.54***	11.23**	39.96***	29.02***	31.29***
MSB vs. Div. opp.	6.13*	1.68	0.12	6.34*	7.49**
MSB vs. CFV	0.48	27.59***	32.09***	13.15***	4.96*
MSB vs. Uncom. opp.	—		—	11.22**	7.14**
Alienated vs. Div. opp.	15.54***	27.28***	30.74***	8.82**	8.13**
Alienated vs. CFV	69.27***	91.08***	139.66***	60.70***	66.16***
Alienated vs. Uncom. opp.	—	—	—	5.42*	7.55**
Div. Opp. vs. CFV	13.24***	18.68***	30.10***	31.33***	27.23***
Div. Opp. vs. Uncom. opp.	—	—	—	5.42*	0.00
CFV vs. Uncom. opp.	—	—	—	41.77***	28.66***
Sat. with government					
MSB vs. alienated	402.18***	339.44***	315.81***	1171.39***	969.54***
MSB vs. Div. opp.	114.72***	16.84***	61.62***	156.94***	266.21***
MSB vs. CFV	23.21***	110.19***	113.56***	38.71***	11.03***
MSB vs. Uncom. opp.	—	—	—	262.28***	84.15***
Alienated vs. Div. Opp.	100.22***	219.69***	61.62***	397.47***	121.59***
Alienated vs. CFV	994.97***	1009.47***	1021.78***	1205.67***	1372.31***
Alienated vs. Uncom. opp.	—	—	—	300.96***	437.98***
Div. Opp. vs. CFV	330.05***	232.64***	348.19***	279.41***	419.31***
Div. Opp. vs. Uncom. opp.	—	—	—	8.98**	60.79***
CFV vs. Uncom. opp.	—	—	—	419.34***	193.08***
Sat. with democracy					
MSB vs. alienated	309.45***	303.84***	282.50***	1127.10***	837.94***
MSB vs. Div. opp.	60.50***	19.89***	34.79***	144.84***	220.96***
MSB vs. CFV	29.13***	51.32***	76.51***	17.82***	12.77***
MSB vs. Uncom. opp.	—	—	—	212.28***	65.32***
Alienated vs. Div. opp.	126.80***	192.79***	93.05***	373.16***	125.48***
Alienated vs. CFV	894.89***	749.86***	830.91***	839.49***	1161.01***
Alienated vs. Uncom. opp.	—	—	—	295.24***	389.47***
Div. Opp. vs. CFV	252.87***	158.22***	215.83***	173.66***	359.47***
Div. Opp. vs. Uncom. opp.	—	—	—	4.38*	50.65***
CFV vs. Uncom. opp.	—	—	—	240.39***	160.96***
EU unification					
MSB vs. alienated	1.60	4.17*	12.88**	4.69*	5.04*
MSB vs. Div. opp.	0.00	0.73	6.40*	14.84***	1.53
MSB vs. CFV	0.01	20.99***	6.68*	9.76**	0.02
MSB vs. Uncom. opp.	—	—	—	12.16***	13.41***
Alienated vs. Div. opp.	2.08	1.73	0.44	3.18	15.63***
Alienated vs. CFV	2.56	10.55**	0.26	3.66	4.99*
Alienated vs. Uncom. opp.	—	—	—	1.75	3.90*
Div. Opp. vs. CFV	0.02	16.82***	0.01	0.95	1.02
Div. Opp. vs. Uncom. opp.	—	—	—	0.22	31.01***
CFV vs. Uncom. opp.	—	—	—	1.53	14.43***
Years of education (self-rep.)					
MSB vs. alienated	2.20	3.66	2.50	0.09	0.91
MSB vs. Div. opp.	10.91***	29.58***	23.95***	7.25**	20.57***
MSB vs. CFV	16.74***	8.38**	26.04***	7.00**	8.50**
MSB vs. Uncom. opp.	—	—	—	4.55*	1.08
Alienated vs. Div. opp.	30.50***	20.93***	13.34***	14.89***	17.36***
Alienated vs. CFV	43.60***	1.81	14.49***	9.33**	5.86*
Alienated vs. Uncom. opp.	—	—	—	6.10*	3.52
Div. Opp. vs. CFV	1.00	9.07**	0.01	1.27	2.74
Div. Opp. vs. Uncom. opp.	—	—	—	37.60***	24.07***
CFV vs. Uncom. opp.	—	—	—	17.51***	13.23***

MSB, moderate system believers; CFV, committed Fidesz voters; Div. opp., diverse opposition; Uncom. opp., uncommitted opposition; Sat. with government, satisfaction with government; Sat. with democracy, satisfaction with democracy. *p < 0.05, **p < 0.01, ***p < 0.001.

With regards to age, Moderate system believers were older than other classes on average (*M*_age_ = 52.2, 54.3, 51.9, 53.3, and 53.8, for the five rounds, respectively), whereas Diverse opposition members were youngest on average (*M*_age_ = 47.6, 48.6, 49.9, 47.9, and 47.5 years, respectively), with Alienated, Committed Fidesz voters, and Uncommitted Opposition falling in between. In 2018, no age differences between classes were found; in other rounds, MSB and Diverse Opposition differed significantly, with some other smaller differences between other classes as well (see Table 3).

Regarding self-reported years of education, Committed Fidesz voters (*M* = 13.2, 12.0, 13.0, 13.6, and 12.5 for the five rounds, respectively) and Diverse Opposition (*M* = 12.9, 12.8, 13.0, 13.0, and 12.9) tended to report highest number of years of education, and Uncommitted Opposition the lowest (*M* = 11.5 and 11.4 for 2020 and 2023, respectively). As shown in Table 3, Diverse Opposition members and Committed Fidesz voters had significantly more years of education than the Moderate System Believers, Alienated, and Uncommitted Opposition members in all rounds (except for CFV vs. Alienated in 2018). In summary, both Committed Fidesz voters and Diverse opposition members consistently reported more years of education than Moderate system believers, Alienated, and Uncommitted Opposition respondents across all years, highlighting clear educational differences between these groups.

The results for binary covariates are presented in Supplementary Table S3. As shown there, first, the Moderate system believers had the highest proportion of women in the 2014–2018 rounds, and the Diverse opposition had highest proportion of men. In 2020, no differences were found in class-specific gender distributions, and in 2023, the Uncommitted opposition class had the highest proportion of women, and Diverse Opposition again the highest proportion of men.

Regarding collective action, Committed Fidesz voters reported engaging in collective action with much higher rates than other classes in 2014 and 2020. Diverse opposition reported high collective action engagement across the rounds and Alienated and Uncommitted opposition reported low rates.

The relationship between class membership and domicil was investigated with chi-square and chi-square post-hoc tests crossing most likely class membership with area, and testing over- or underrepresentation against sample proportions. The domicil category *farm/countryside home* was dropped from these comparisons because of low number of respondents reporting this area of residence (0 to 17 respondents across rounds), and suburb/outskirts of a town was additionally dropped from the last two rounds for low frequencies in this category for those rounds (< 10 respondents). The results were variable, but Diverse Opposition and Committed Fidesz voters tended to be overrepresented among city residents and underrepresented among country village residents, and the opposite was true of the Alienated and Uncommitted opposition.

## 4 Discussion

The present study investigated Hungarian citizens’ system-related beliefs and attitudes using latent profile analysis during the late Orbán/Fidesz period. The resulting profiles were examined primarily in relation to institutionalized and non-institutionalized political participation, and secondarily in relation to satisfaction with the status quo, happiness, attitudes toward European unification, and demographic characteristics. While accounts of Hungarian politics have emphasized strong elite polarization and political tribalism (e.g., Bíró-Nagy et al., 2022; Krekó, 2021), previous research has already suggested that this picture is incomplete, pointing to the heterogeneous social and attitudinal bases of Fidesz support and opposition (Scoggins, 2022; Szabó et al., 2021; Wunsch and Gessler, 2023). Our findings add to this literature by showing that citizens’ psychological-political orientations did not neatly align with simple categories of system supporters, system critics, and passive bystanders. Instead, the analysis consistently revealed more differentiated profiles, including Moderate system believers, Committed Fidesz voters, Alienated citizens, and Diverse opposition members.

These results qualify, rather than refute, accounts of polarization and political tribalism in Hungary. They suggest that such accounts may capture important features of elite conflict and public discourse, while still overlooking heterogeneity in how citizens related to the political system. The findings also have broader relevance beyond Hungary, especially because the Hungarian model was at times explicitly invoked abroad as a model of statecraft (Applebaum, 2025). Finally, read in light of Fidesz’s 2026 electoral defeat and the end of Orbán’s 16-year rule, the results suggest that the social-psychological foundations of the regime were more fragmented and contingent than narratives of a uniformly tribalized electorate would imply.

### 4.1 Citizen-level perspectives on the Hungarian political system

One of our main findings concerning Hungarian citizens’ political orientations is that voters of the governing party constituted a heterogeneous group in terms of political interest, self-efficacy, institutional trust, and left–right ideological self-placement. Fidesz supporters appeared in every class, including, somewhat surprisingly, the Diverse opposition group.

However, these voters were primarily concentrated in two classes: the Moderate system believers and Committed Fidesz voters. The former represents a larger group characterized by moderate political interest, high institutional trust, low self-efficacy, and centrist ideological views. The latter, by contrast, constitutes a smaller but more ideologically committed group marked by very high levels of interest, trust, and self-efficacy, along with a strong right-wing orientation. Both groups expressed satisfaction with the status quo, yet they diverged in their attitudes toward European unification: Moderate system believers were considerably more supportive of EU integration, whereas Committed Fidesz voters were more skeptical. These findings support Wunsch and Gessler’s (2023) argument that Fidesz’s success rested on uniting a diverse coalition rather than mobilizing a single, coherent tribe. Significantly, they also suggest that many Fidesz voters maintained moderate ideological views and pro-EU attitudes despite the government’s increasingly sovereignty-based critique of the European Union (Hargitai, 2020; Scoggins, 2022). These voters expressed trust in institutions, yet believed that neither they nor other citizens could meaningfully influence the political system—a finding that appears paradoxical, given that most members of this group supported the party that governed for over a decade. By contrast, the smaller, ideologically committed group appears to have aligned more closely with the governing party’s positions, including its confrontational stance toward the European Union.

Another significant finding is the distinction between passive and opposition voters. Most notably, opposition voters consistently displayed higher levels of institutional trust and self-efficacy in each round compared to the passive group, and this translated into greater political engagement—both in institutionalized, legal forms and in non-institutionalized, legal forms. This underscores the importance of maintaining at least some trust in the system to motivate political engagement, with self-efficacy possibly acting as a mediator between the two (Cichocka et al., 2018; Osborne et al., 2019).

This finding carries a crucial implication for those seeking change: opposition politicians should avoid entirely delegitimizing the system, as doing so may demobilize people rather than mobilize them. There has been an intense debate among opposition politicians about whether to mock the system and deny the legitimacy of its institutions. For instance, after the loss in the 2022 parliamentary elections, some opposition figures argued that opposition MPs should boycott parliament, claiming that the election was fraudulent and that the newly formed parliament had lost its democratic function. Others, however, contended that change could not be achieved without working within the existing framework (Horváth, 2022).

Our results align more closely with the arguments of the latter group. This is not a trivial finding, as debates about the “rotten” nature of the system were central to opposition strategy during the later Orbán/Fidesz period. However, it appears that extreme system derogation was more strongly associated with demobilization than with perceiving the establishment as something against which meaningful resistance was possible. Similar findings were reported by Benza et al. (2025), who found that independent media portrayals of the system as irreparably corrupt had a demobilizing effect on their audiences. Importantly, this is not a debate about which narrative is “right” from the opposition’s point of view. Instead, our results suggest that, among citizens, political participation was more characteristic of those who retained some trust in the system and a belief in their own political efficacy.

This aligns with traditional arguments in social identity theory, which posit that without a cognitive alternative—a viable, conceivable way for the status quo to be changed—disadvantaged members of society are more likely to accept the system and avoid direct competition or protest (Tajfel and Turner, 1979).

This insight is crucial: the more lethargic Fidesz’s political opponents become, the less chance they have of challenging the system. Earlier versions of this manuscript noted only the emergence of a new political opponent of Fidesz, led by a former insider, and suggested that such a movement might mobilize previously alienated individuals by offering a more credible alternative. Subsequent developments have made that interpretation more compelling. In the 2026 parliamentary election, Péter Magyar’s Tisza defeated Fidesz and won a two-thirds parliamentary majority, ending Orbán’s 16-year rule. Although our data do not extend to the 2026 election and therefore cannot explain that outcome directly, the speed and scale of this realignment are difficult to reconcile with earlier portrayals of Hungary as a system locked in durable, self-reinforcing stability. Instead, they suggest that even entrenched systems may contain latent potential for sudden breaks when new cognitive alternatives become salient and credible.

The question of democratic backsliding is closely tied to the citizen-level patterns discussed in this study. Among scholars, there is broad consensus that Hungary underwent significant democratic erosion. Consequently, participants’ responses are often interpreted from the assumed premise that democracy was in decline. However, it remains unclear whether citizens were fully aware of this backsliding, or whether they perceived it as such (Chapman et al., 2024). Wunsch and Gessler (2023) argue that tolerance of democratic decline is partly due to partisans prioritizing perceived benefits over democratic norms. Yet they also highlight that citizens may fail to recognize violations, or may operate with alternative conceptions of democracy, leading to divergent interpretations of political developments.

Scheppele (2022) similarly notes that Fidesz implemented reforms that, when viewed in isolation, appeared procedurally acceptable, but cumulatively dismantled liberal democratic institutions. Vachudova (2021) emphasizes the difficulty of measuring democratic backsliding, raising the critical question of where ordinary political behavior ends and authoritarian erosion begins. Public opinion data further suggest that government and opposition voters assessed political developments through starkly different lenses. For example, Csehi (2019) reports that Fidesz voters supported an electoral system that overcompensated the winner if it ensured governmental stability—even at the expense of proportional representation. Moreover, only Fidesz voters viewed the government as having the legitimacy to draft a new constitution without opposition input.

Importantly, when government voters’ decisions are interpreted through a tribalistic lens, they are often seen as sacrificing democratic norms in favor of partisan loyalty. However, it remains unclear whether these voters actually perceived democratic backsliding. If not, their decisions may have been more moderate and interest-based than assumed, a possibility supported by the larger, more moderate government-supporting class identified in our analysis. The 2026 defeat of Fidesz reinforces this point indirectly: if a substantial portion of the governing party’s electorate had indeed been bound primarily by pragmatic or contingent support rather than hardened tribal loyalty, the coalition may have been more vulnerable to rapid reconfiguration once a credible challenger emerged.

### 4.2 Broader implications of electoral autocracy: the normalization and export of the Hungarian model

The Hungarian case is important not only as a national example of democratic backsliding, but also because the Orbán/Fidesz system became a broader political reference point beyond Central and Eastern Europe. During a 2021 visit to Budapest, Tucker Carlson portrayed Orbán’s Hungary as a model of political “order and clarity” (Smith, 2021), and during the 2024 United States presidential debate, Donald Trump referred to Orbán as “one of the most respected men” and a “smart prime minister of Hungary” (Ordoñez, 2024). In a similar vein, Applebaum (2025) recalled the 2022 CPAC Dallas conference, where Kevin Roberts of the Heritage Foundation described Hungary as “not just as a model for modern statecraft, but the model.” More broadly, commentators have argued that the erosion of American institutions during Trump’s second presidency resembled some of the legislative changes introduced at the beginning of the Orbán period (Spike and Riccardi, 2025), while the rhetoric of the two leaders has been noted to share features of hijacked victimhood, including the reversal of victim and victimizer roles, the construction of new moral orders, the teleological framing of past and future harms, and the mobilization of security threats to preserve or expand power (Hronešová and Kreiss, 2024). This broader relevance raises an important question: how did such a system shape citizens’ system-related attitudes and beliefs—not only within Hungary, but also as a case with implications for other societies moving in a similar direction?

Apparently, a large segment of the governing party’s own supporters lacked a sense of political efficacy. Speculatively, their voting behavior may have been driven more by economic considerations (see Scoggins, 2022) and by a lack of a cognitive alternative. In parallel, there existed a segment of the opposition characterized by diminished trust and efficacy; a form of political lethargy observable in how this group changed over the five rounds of the European Social Survey. Additionally, a sizable portion of Hungarian citizens felt unrepresented, alienated from both government and opposition parties.

Elite-level polarization and a tribalistic political culture in Hungary therefore did not translate into uniformly polarized citizen profiles. Instead, they coincided with a fatigued population characterized by low political efficacy and only low to moderate levels of institutional trust. In a “megastudy” testing 25 different interventions to reduce antidemocratic attitudes and partisan animosity, one of the more effective treatments—particularly in reducing partisan animosity—was the common exhausted majority identity intervention, which emphasized that political divisions are amplified by the media and that most people are, in fact, tired of polarization (Voelkel et al., 2024).

A similar dynamic may have unfolded in Hungary, where political parties, elite rhetoric, and the media were deeply polarized (Benza et al., 2025). Yet beneath this surface-level polarization lay a broader and more troubling pattern: a tired and alienated population, coexisting with smaller but more politically active factions. This configuration carries an implicit warning for supporters of modern liberal democracies. It may be comforting to see Hungary’s democratic backsliding as a unique case: a small and fragile post-communist state, captivated by a charismatic leader after the moral collapse of a discredited social democratic government, and further destabilized by the global financial crisis. Hungary would thus be just another country in which externally encouraged democratization, in the absence of sufficient internal foundations, leads to stagnation or reversal (Diskin et al., 2005; Ekiert et al., 2007).

However, the data tell a more unsettling story—one that could just as easily unfold in long-established democracies. A political party opportunistically seized power during a period of moral and economic decline, reshaped institutional rules to entrench its power, cultivated a group of devoted supporters, and built a coalition that included not only ideologically committed supporters, but also moderate, centrist voters motivated by economic pragmatism or a perceived lack of alternatives. Meanwhile, growing segments of the population felt unrepresented or powerless, with only a shrinking minority believing in the possibility of meaningful opposition. Similar patterns have emerged in established democracies such as the United States, where democratic norms have eroded through election denialism, institutional manipulation, and the normalization of political violence, particularly during and after the Trump presidency (Grumbach, 2022; Levitsky and Ziblatt, 2018). The danger lies in using Hungary’s trajectory not as a cautionary tale but as a blueprint for modern statecraft (see also Applebaum, 2025). At the same time, the 2026 Hungarian election suggests that even systems long perceived as deeply entrenched may remain politically reversible, though not without difficulty. The persistence of Orbán-era institutional arrangements and loyalist appointments indicates that electoral defeat does not automatically undo the broader legacy of democratic erosion.

### 4.3 Researcher positionality and interpretive inflation in narratives of polarized tribes

This section speaks to a broader issue: how dominant political narratives can shape empirical interpretations and reinforce perceived polarization even when the data suggest a more nuanced reality. While this study set out to examine citizen-level correlates and consequences of political attitudes within the context of Hungary’s macro-level transformation, the findings diverged, at first glance, from some influential interpretations in the existing literature. Previous studies have framed the Hungarian context in terms of deeply divided political tribes (Krekó, 2021), high levels of collective narcissism (Lantos and Forgas, 2021), and cognitively rigid right-wing voters justifying the status quo (Jost and Kende, 2020). These studies capture important aspects of Hungarian politics and identify theoretically meaningful relationships. However, a closer inspection of the underlying data suggests a more nuanced picture of what Enyedi and Krekó (2018) have called “Orbán’s laboratory of illiberalism.” In this laboratory, what was being synthesized may not have been a population split into radically distinct ideological camps, but rather the illusion of such a division.

Researcher positionality—the influence of scholars’ own ideological orientations on their interpretations—has long been recognized as a source of bias in social science (e.g., Harding, 1991). In contexts such as Hungary, where public discourse is highly polarized, media freedom is restricted, and academic freedom is constrained, even well-intentioned researchers are not merely external observers of politics. They may also become involved in symbolic struggles over the legitimate “vision and division” of society, to borrow from Bourdieu’s framework (Bourdieu, 1985, 1989). In such settings, how society is described can become part of the broader political struggle itself, and may inadvertently reinforce binary narratives.

For instance, Krekó’s (2021) study used Hungary to illustrate how tribalism was cultivated and sustained through elite rhetoric, media control, and identity-based mobilization. However, a closer look at the numbers in that study shows that government supporters scored slightly below the midpoint on the Manichean worldview scale and reported high levels of pluralism—hardly consistent with an entrenched tribal mindset. Similarly, Lantos and Forgas (2021) emphasized the “pervasive role” of collective narcissism in populist attitudes and voting intentions. Yet most participants in their study disagreed with the collective narcissism items and showed pro-EU attitudes. This does not invalidate the correlation between collective narcissism and pro-government voting, but it does raise questions about the scope and centrality of this explanatory variable. Likewise, Jost and Kende (2020) reported a small correlation between cognitive rigidity and right-wing political orientation in a large sample—suggesting only a trivial association. They also found moderate levels of system justification among government supporters and relatively high economic system justification among opposition voters. These findings challenge the narrative of sharply opposed ideological groups. In the same vein, Lönnqvist et al. (2021) found that system justification increased between 2010 and 2018 across both camps, pointing to a general rise in perceived legitimacy—but not to extreme levels, even among Fidesz supporters.

Why, then, do such discrepancies persist between empirical findings and dominant narratives? One possible explanation lies in researcher positionality, that is, the biases and ideological commitments of researchers themselves. As Bilewicz et al. (2015) noted, many social and political psychologists in Hungary lean toward liberal or left-wing orientations. This may inadvertently contribute to interpretive biases, particularly in framing support for Fidesz as a product of being misled by propaganda or lacking critical capacity. Such framing reduces complex political choices to a moral binary and risks reinforcing the “good versus evil” narrative. This perspective overlooks the possibility that many Fidesz voters made pragmatic, interest-based choices—for example, prioritizing economic security—without endorsing the full ideological platform of the governing party. Moreover, the widely circulated stereotype of the “typical” Fidesz voter as an older, less educated, rural male is not strongly supported by our data.

At the same time, discrepancies between empirical findings and dominant narratives do not need to be explained by researcher positionality alone. They may also reflect conventional interpretive practices in social and political psychology, where statistically significant but substantively modest associations are often discussed as theoretically meaningful differences between groups. In politically charged contexts, such reporting conventions may contribute to interpretive inflation, especially when relative differences are translated into categorical claims about citizens or political camps. Researcher positionality and methodological convention are therefore not mutually exclusive explanations; separately or jointly, they may shape which empirical patterns become salient and how strongly they are interpreted.

This critique is therefore not aimed at individual scholars, but rather at the broader tendency of dominant narratives to shape interpretation, especially in polarized political contexts. When such narratives become self-reinforcing, they risk distorting the data and deepening perceived divides between imagined groups of “us” and “them.” The danger is that moderate citizens may be mischaracterized as tribalistic, thereby reinforcing the very polarization such narratives seek to explain. The 2026 defeat of Fidesz makes this point even more salient. If the Hungarian electorate had in fact been fully locked into rigid, mutually exclusive political tribes, the scale and rapidity of the electoral realignment would have been harder to account for. This does not mean polarization was absent, but it does suggest that dominant narratives may at times have overstated its social depth. Our findings therefore underscore the need for greater reflexivity in political psychology and a stronger commitment to empirical nuance.

### 4.4 Limitations

There are several notable limitations to the present study. First, as previously mentioned, there is a degree of arbitrariness in determining what constitutes a grouping variable in LPA versus what should be treated as a correlate. Second, the ESS imposes constraints on the types of research questions that can be pursued. In particular, the range of available items limits the explanatory power regarding political participation. For example, while the Social Identity Model of Collective Action (SIMCA) offers a useful theoretical framework (van Zomeren et al., 2008), it can only be partially applied in this context, as key components such as emotions and moral convictions are not measured in the ESS. Other important determinants of political participation are similarly missing, including perceptions of political corruption—a factor central to discussions about the Hungarian political system (Enyedi and Krekó, 2018) and widely recognized as a demobilizing force through mechanisms such as “exiting the system” (Snegovaya, 2020). Furthermore, the ESS captures only legal forms of collective action. Given that instances of illegal or non-institutionalized political participation have occurred in the Hungarian context, it would be important to assess the willingness of different latent classes to engage in such actions—an aspect that remains beyond the scope of this dataset.

A further limitation is temporal. Our data end in 2023/24 and therefore do not include the political developments culminating in the 2026 election. These developments can inform the interpretation of our findings, but they do not constitute evidence generated by the present analyses. Future research should therefore examine whether the profiles identified here persisted, transformed, or realigned in response to the emergence of a newly credible opposition and the end of Fidesz’s long period in power.

## 5 Conclusion

This study advances a more nuanced understanding of Hungary’s political system by shifting attention from macro-level institutional transformation to citizen-level psychological and behavioral patterns. Our person-centered analysis identified diverse profiles of political engagement and disengagement, challenging the notion of a uniformly tribalized electorate. Both government and non-government supporters appeared across distinct engagement types, suggesting a more fragmented and dynamic landscape than often assumed.

These findings have both theoretical and practical implications. Theoretically, they call into question dominant interpretations within political psychology that rely on binary narratives of tribalism and ideological rigidity. Practically, they underscore the importance of trust and efficacy in motivating political participation, particularly for those seeking change within systems perceived as unfair or unresponsive. Moreover, Hungary’s case offers lessons for other democracies showing signs of democratic decline: illiberal consolidation may not require mass polarization, only the gradual erosion of trust, efficacy, and viable alternatives.

Finally, this study raises important methodological and epistemological concerns about the relationship between data and interpretation. Dominant narratives can diverge significantly from empirical evidence. As illiberal models gain international attention, the need for accurate, nuanced, and reflexive scholarship becomes all the more urgent. The 2026 defeat of Fidesz further underscores that even long-standing illiberal systems may rest on more fragile and heterogeneous social-psychological foundations than accounts of entrenched tribalism suggest. Orbán’s fall after 16 years in power does not invalidate analyses of democratic erosion in Hungary; rather, it highlights that such systems can appear durable while remaining vulnerable to rapid reconfiguration once a credible alternative emerges.

## Data Availability

Publicly available datasets were analyzed in this study. This data can be found here: The raw data supporting the conclusions of this article, along with the R code and MPlus code used for data analysis, are available on the Open Science Framework: https://osf.io/mky93/?view_only=3400f94ba1834393a640ecb754e6edc8.

## Footnotes

1
https://www.europeansocialsurvey.org
