# Supplementary material for: Political participation in an electoral autocracy: latent profiles of efficacy, interest, ideology, and trust in Hungary
Source: Front Psychol. 2026 Jul 30;17:1856578. doi: 10.3389/fpsyg.2026.1856578 (PMC13469075; doi:10.3389/fpsyg.2026.1856578)
Supplement: Supplementary file 1 [file Table_1.docx]

| Table S1. Party voted in the last national election, percentages out of those eligible to vote who reported either the party voted or that they did not vote. | | | | | |
| --- | --- | --- | --- | --- | --- |
|  | **2014** | **2016** | **2018** | **2020** | **2023** |
| Fidesz | 32.7 % | 46.5 % | 39.0 % | 39.6 % | 44.9 % |
| MSZP | 17.5 % | 13.0 % | 8.0 % | 6.0 % | - |
| Jobbik | 12.5 % | 8.1 % | 9.1 % | 9.1 % | - |
| LMP | 3.8 % | 1.5 % | 3.5 % | 3.4 % | - |
| Munk | 0.05 % | 0.4 % | - | - | - |
| DK | - | - | 5.1 % | 8.7 % | - |
| KDNP | - | - | 1.7 % | - | - |
| Momentum | - | - | 1.4 % | 3.6 % | - |
| Ellenzéki-Öss. | - | - | - | - | 22.4 % |
| Mi Hazank | - | - | - | - | 5.5 % |
| MKKP | - | - | - | - | 1.3 % |
| Did not vote | 33.1 % | 30.4 % | 32.1 % | 29.0 % | 25.9 % |
| *Note*. Fidesz = Fidesz – Magyar Polgári Szövetség (Fidesz – Hungarian Civic Alliance); MSZP = Magyar Szocialista Párt (Hungarian Socialist Party); Jobbik = Jobbik Magyarországért Mozgalom (Movement for a Better Hungary); LMP = Lehet Más a Politika (Politics Can Be Different); Munk = Magyar Munkáspárt (Hungarian Workers’ Party); DK = Demokratikus Koalíció (Democratic Coalition); KDNP = Kereszténydemokrata Néppárt (Christian Democratic People’s Party); Momentum = Momentum Mozgalom (Momentum Movement); Ellenzéki-Öss. = Ellenzéki Összefogás (Opposition Alliance); Mi Hazánk = Mi Hazánk Mozgalom (Our Homeland Movement); and MKKP = Magyar Kétfarkú Kutya Párt (Hungarian Two-Tailed Dog Party). Only participants who reported either the party they voted or that they did not vote in the last election were included. In addition, respondents reporting not being eligible to vote (*N*s = 86, 101, 76, 126, and 77 for 2014, 2016, 2018 2020, and 2023 respectively) and respondents who voted for a party with less than 15 voters in total in that round were removed (*N*s = 0, 5, 24, 24, and 22 for 2014, 2016, 2018 2020, and 2023 respectively). This resulted in the following *N*s for the voting behavior analysis: *N*=1277 (75 % of the total sample) for 2014, *N*=1246 (77 %) for 2015, *N*=1331 (80 %) for 2018, *N*=1432 (77 %) for 2020, and *N* = 1740 (82 %) for 2023). | | | | | |
