# Supplementary material for: Political participation in an electoral autocracy: latent profiles of efficacy, interest, ideology, and trust in Hungary
Source: Front Psychol. 2026 Jul 30;17:1856578. doi: 10.3389/fpsyg.2026.1856578 (PMC13469075; doi:10.3389/fpsyg.2026.1856578)
Supplement: Supplementary file 2 [file Table_2.docx]

| Table S2. Percentages of voting a particular party and not voting per latent class. | | | | | |
| --- | --- | --- | --- | --- | --- |
|  | ”Moderate system believers” | ”Committed Fidesz voters” | ”Alienated” | ”Diverse opposition” | ”Uncommitted opposition” |
| **2014** |  |  |  |  |  |
| Fidesz | 50.2 % | 57.5 % | 20.9 % | 18.2 % |  |
| MSZP | 10.0 % | 16.7 % | 16.1 % | 26.8 % |  |
| Jobbik | 6.8 % | 11.9 % | 10.9 % | 20.4 % |  |
| LMP | 3.2 % | 5.6 % | 2.5 % | 5.0 % |  |
| Munk | 1.0 % | 0 | 0.02 % | 1.1 % |  |
| Did not vote | 29.0 % | 8.3 % | 49.3 % | 28.6 % |  |
| **2016** |  |  |  |  |  |
| Fidesz | 50.4 % | 80.0 % | 22.8 % | 46.5 % |  |
| MSZP | 10.7 % | 3.8 % | 17.0 % | 17.4 % |  |
| Jobbik | 5.4 % | 6.0 % | 8.4 % | 11.1 % |  |
| LMP | 1.2 % | 1.2 % | 1.0 % | 2.9 % |  |
| Munk | 0.4 % | 0 | 0.5 % | 0.6 % |  |
| Did not vote | 32.2 % | 8.8 % | 51.1 % | 22.1 % |  |
| **2018** |  |  |  |  |  |
| Fidesz | 43.3 % | 77.0 % | 15.5 % | 34.4 % |  |
| MSZP | 8.3 % | 2.4 % | 10.2 % | 9.6 % |  |
| Jobbik | 6.0 % | 4.8 % | 11.6 % | 13.1 % |  |
| LMP | 3.7 % | 0.8 % | 3.9 % | 5.2 % |  |
| DK | 5.7 % | 1.2 % | 4.8 % | 8.2 % |  |
| KDNP | 2.3 % | 2.8 % | 1.2 % | 0.7 % |  |
| Momentum | 1.0 % | 0.8 % | 1.5 % | 2.7 % |  |
| Did not vote | 30.2 % | 10.3 % | 51.3 % | 9.6 % |  |
| **2020** |  |  |  |  |  |
| Fidesz | 69.4 % | 79.4 % | 16.5 % | 36.9 % | 30.4 % |
| MSZP | 0.7 % | 0.9 % | 8.4 % | 6.4 % | 9.0 % |
| Jobbik | 6.3 % | 7.5 % | 9.6 % | 11.9 % | 9.0 % |
| LMP | 1.5 % | 0.9 % | 5.1 % | 3.7 % | 3.8 % |
| DK | 4.0 % | 1.9 % | 12.0 % | 10.2 % | 9.7 % |
| Momentum | 2.2 % | 0.9 % | 4.2 % | 5.4 % | 3.5 % |
| Did not vote | 15.8 % | 8.4 % | 44.3 % | 25.4 % | 34.7 % |
| **2023** |  |  |  |  |  |
| Fidesz | 68.5 % | 73.4 % | 18.2 % | 24.5 % | 53.1 % |
| Ellenzéki Ö. | 12.6 % | 9.5 % | 30.5 % | 39.5 % | 16.2 % |
| Mi Hazank | 2.3 % | 7.5 % | 5.3 % | 10.0 % | 2.5 % |
| MKKP | 0.7 % | 2.0 % | 1.3 % | 1.9 % | 0.8 % |
| Did not vote | 15.9 % | 7.5 % | 44.7 % | 24.1 % | 27.3 % |
| Note. Fidesz = Fidesz – Magyar Polgári Szövetség (Fidesz – Hungarian Civic Alliance); MSZP = Magyar Szocialista Párt (Hungarian Socialist Party); Jobbik = Jobbik Magyarországért Mozgalom (Movement for a Better Hungary); LMP = Lehet Más a Politika (Politics Can Be Different); Munk = Magyar Munkáspárt (Hungarian Workers’ Party); DK = Demokratikus Koalíció (Democratic Coalition); KDNP = Kereszténydemokrata Néppárt (Christian Democratic People’s Party); Momentum = Momentum Mozgalom (Momentum Movement); Ellenzéki-Öss. = Ellenzéki Összefogás (Opposition Alliance); Mi Hazánk = Mi Hazánk Mozgalom (Our Homeland Movement); and MKKP = Magyar Kétfarkú Kutya Párt (Hungarian Two-Tailed Dog Party). Percentages of voters by class and party voted / not voting are shown. For each round, parties that less than 15 respondents in the whole sample reported voting for were omitted. | | | | | |
