# Supplementary material for: Political participation in an electoral autocracy: latent profiles of efficacy, interest, ideology, and trust in Hungary
Source: Front Psychol. 2026 Jul 30;17:1856578. doi: 10.3389/fpsyg.2026.1856578 (PMC13469075; doi:10.3389/fpsyg.2026.1856578)
Supplement: Supplementary file 3 [file Table_3.DOCX]

| Table S3. Categorical covariates’ proportions and results of chi-square tests for the differences between classes. | | | | | |
| --- | --- | --- | --- | --- | --- |
|  | 2014 | 2016 | 2018 | 2020 | 2023 |
| Gender |  |  |  |  |  |
| **Sample** | **57 % women** | **58 % women** | **57 % women** | **62 % women** | **61 % women** |
| MSB | 64 % women | 65 % women | 65 % women | 63 % women | 63 % women |
| Alien. | 60 % women | 56 % women | 61 % women | 61 % women | 58 % women |
| Div.O. | 50 % women | 52 % women | 49 % women | 60 % women | 56 % women |
| CFV | 54 % women | 62 % women | 50 % women | 59 % women | 62 % women |
| Un.Opp. | - | - | - | 65 % women | 65 % women |
| χ^2^ results | MSB > DO***  MSB > CFV*  A > DO** | MSB > DO***  MSB > A***  CFV > DO** | MSB > DO, CFV***  A > CFV** | No significant differences between classes | UO > DO, A** |
| Voting**^1^** |  |  |  |  |  |
| **Sample** | **74% voted** | **75 % voted** | **73 % voted** | **75 % voted** | **78 % voted** |
| MSB | 78 % voted | 75 % voted | 74 % voted | 86 % voted | 86 % voted |
| Alien. | 62 % voted | 59 % voted | 61 % voted | 65 % voted | 63 % voted |
| Div.O. | 77 % voted | 82 % voted | 78 % voted | 78 % voted | 79 % voted |
| CFV | 93 % voted | 92 % voted | 90 % voted | 92 % voted | 93 % voted |
| Un.Opp. | - | - | - | 73 % voted | 77 % voted |
| χ^2^ results | CFV > others***  MSB > A** | CFV > A, MSB***  CFV > DO**  DO > A***  DO > MSB**  MSB > A** | CFV > MSB, A***  CFV > DO**  DO > A***  MSB > A** | CFV > A, UO, DO***  CFV > MSB*  MSB > A, UO***  MSB > DO*  DO > A***  DO > UO* | CFV > A, DO, UO***  MSB > A, UO***  MSB > DO**  UO > A** |
| Coll. Act. | 2014 | 2016 | 2018 | 2020 | 2023 |
| **Sample** | **16 % yes** | **13 % yes** | **13 % yes** | **17 % yes** | **15 % yes** |
| MSB | 10 % yes | 8 % yes | 6 % yes | 17 % yes | 17 % yes |
| Alien. | 7 % yes | 7 % yes | 7 % yes | 16 % yes | 11 % yes |
| Div.O. | 23 % yes | 25 % yes | 22 % yes | 26 % yes | 22 % yes |
| CFV | 32 % yes | 12 % yes | 25 % yes | 31 % yes | 17 % yes |
| Un.Opp. | - | - | - | 10 % yes | 8 % yes |
| χ^2^ results | CFV > A, MSB***  CFV > DO*  DO > A, MSB*** | DO > others***  CFV > A* | CFV > MSB, A***  DO > MSB, A*** | CFV > UO***  CFV > A, MSB**  DO > UO***  DO > A, MSB**  MSB > UO** | DO > A, UO***  CFV > UO***  CFV > A**  MSB > UO**  MSB > A* |
|  | 2014 | 2016 | 2018 | 2020 | 2023 |
| Liv. area | **City/Suburb/Small town/Country village** | **City/Suburb/Small town/Country village** | **City/Suburb/Small town/Country village** | **City/Suburb/Small town/Country village^2^** | **City/Suburb/Small town/Country village^2^** |
| **Sample** | **26 / 3 / 37 / 33 %** | **28 / 6 / 37 / 29 %** | **21 / 5 / 37 / 37 %** | **26 / 2 / 34 / 39 %** | **25 / 1 / 21 / 53 %** |
| MSB | 25 / 1 / 40 / 33 % | 23 / 5 / 41 / 32 % | 25 / 3 / 35 / 37 % | 23 / 6 / 39 / 32 % | 20 / 0 / 23 / 57 % |
| Alien. | 25 / 3 / 34 / 38 % | 25 / 5 / 41 / 29 % | 19 / 1 / 37 / 42 % | 20 / 1 / 32 / 47 % | 20 / 3 / 22 / 54 % |
| Div.O. | 28 / 5 / 33 / 34 % | 24 / 10 / 36 / 30 % | 17 / 14 / 35 / 35 % | 36 / 1 / 34 / 29 % | 38 / 1 / 17 / 43 % |
| CFV | 28 / 1 / 49 / 22 % | 43 / 2 / 29 / 26 % | 21 / 5 / 44 / 30 % | 39 / 1 / 28 / 32 % | 40 / 0 / 22 / 39 % |
| Un.Opp. | - | - | - | 22 / 1 / 33 / 44 % | 15 / 1 / 19 / 66 % |
| χ^2^ results | CFV- country village less likely***  CFV – small town more likely***  DO - suburb more likely* | CFV – city more likely***  CFV – small town less likely*  DO – suburb more likely*** | A – suburb less likely***  A – country village more likely*  DO – suburb more likely*** | DO – city more likely, country village less likely***  CFV – city more likely**  A – country village more likely**  UO – country village more likely*  MSB – small town more likely* | CFV and DO – city more likely, country village less likely***  UO – city less likely, country village more likely***  A – city less likely* |
| *Note*. ^1^Voting percentage was counted from those eligible to vote (95%, 94 %, 95 %, 93 %, and 96 % of the sample for each round, respectively).  ^2^The “suburb” category was dropped from chi-square tests for 2020 and 2023 because its low frequencies (0 to 19) made the post-hoc chi-square approximation unreliable.  MSB = Moderate System Believers. A / Alien. = Alienated. DO / Div.O. = Diverse Opposition. CFV = Committed Fidesz Voters. UO / Un.Opp. = Uncommitted Opposition. Coll. Act. = Collective Action.  χ^2^ results for gender, voting, and collective action are from the BCH analyses (see text for details).  χ^2^ results for living area are from a chi-square post-hoc test utilizing standardized residuals, and they test over- and underrepresentation in a given living area relative to the sample proportions. The category “farm/countryside home” was dropped from these analyses due to very small number of respondents in this category across rounds (*N*s 3-15). Suburb = Suburb or outskirts of a large city.  *** *p* < .001. ** *p* < .01. * *p* < .05. | | | | | |
